# Fighting in a wasteland: deleterious metabolites and antitumor immunity

**DOI:** 10.1172/JCI148549

**Published:** 2022-01-18

**Authors:** McLane J. Watson, Greg M. Delgoffe

**Affiliations:** 1 Department of Immunology, University of Pittsburgh, Pittsburgh, Pennsylvania, USA.; 2 Tumor Microenvironment Center, Cancer Immunology and Immunotherapy Program, UPMC Hillman Cancer Center, Pittsburgh, Pennsylvania, USA.

## Abstract

As cancers progress, they produce a local environment that acts to redirect, paralyze, exhaust, or otherwise evade immune detection and destruction. The tumor microenvironment (TME) has long been characterized as a metabolic desert, depleted of essential nutrients such as glucose, oxygen, and amino acids, that starves infiltrating immune cells and renders them dysfunctional. While not incorrect, this perspective is only half the picture. The TME is not a metabolic vacuum, only consuming essential nutrients and never producing by-products. Rather, the by-products of depleted nutrients, “toxic” metabolites in the TME such as lactic acid, kynurenine, ROS, and adenosine, play an important role in shaping immune cell function and cannot be overlooked in cancer immunotherapy. Moreover, while the metabolic landscape is distinct, it is not unique, as these toxic metabolites are encountered in non-tumor tissues, where they evolutionarily shape immune cells and their response. In this Review, we discuss how depletion of essential nutrients and production of toxic metabolites shape the immune response within the TME and how toxic metabolites can be targeted to improve current cancer immunotherapies.

## Introduction

Cancer immunotherapy has revolutionized the treatment of cancer, unlocking the door to durable disease-free states in a subset of patients. Accompanying this major advancement is an improved understanding of the interaction of the immune system and cancer as well as major barriers preventing successful antitumor immunity. One such barrier is the harsh metabolic landscape of the tumor microenvironment (TME). It is well appreciated that tumor cells are metabolically deranged (1), resulting in a hypoxic, acidic, glucose- and amino acid–deprived environment. Many tumors undergo “Warburg metabolism,” or aerobic glycolysis — the process of performing glycolysis despite the presence of adequate oxygen — to meet their biosynthetic and energetic needs (2–4). In addition, rapid proliferation and aberrant cell signaling result in inadequate vasculature and thus poor oxygenation of the TME (more on how tumor cells deplete metabolites can be found in refs. 5, 6). Many have investigated and discussed how the lack of these metabolites inhibits infiltrating effector immune cells, such as NK cells, macrophages, and CD8^+^ and CD4^+^ T cells. While these perspectives are incredibly important, the tumor is not a metabolic vacuum that only consumes and never produces. Therefore, while tumor cells deplete glucose, oxygen, and amino acids, the metabolites they produce are equally, if not more, important in shaping immune cell function and response to immunotherapy. The rules of the game are changing as technology and methods allow us to probe deeper into the true interactions between immune cell metabolism and the TME in vivo. We can no longer rely on the simple model of tumors starving immune cells but must also consider the impact of the “toxic” catabolites produced in shaping immune cell function. Moreover, the metabolic landscape of the TME, while distinct, is not unique, as catabolites such as lactic acid, kynurenine, adenosine, and reactive oxygen species (ROS) are regularly encountered in various tissues and immunologic contexts. It is these non-tumor contexts that have evolutionarily shaped the immune cell–catabolite interactions that play out in the TME. Therefore, it is important to view the TME as one of many metabolic contexts in which immune cells may find themselves and to seek metabolic insight about tumor-infiltrating lymphocytes from non-tumor contexts. This perspective will be crucial in implementing metabolic strategies to improve immunotherapy, as it will elucidate how these therapies may impact typical immunity.

**Figure 1:**
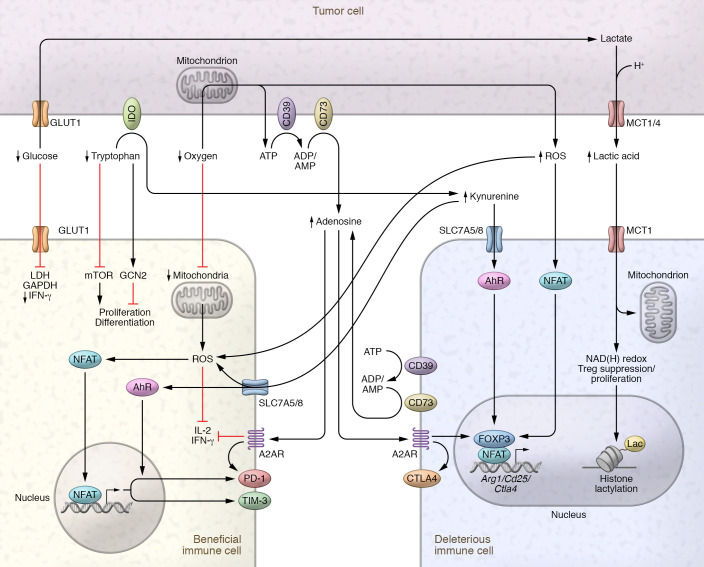
Depletion of key nutrients and production of toxic by-products impair effector cells but support regulatory cells. Beneficial cells, such as CD8^+^ and CD4^+^ effector T (Teff) cells, are depicted at left, and deleterious cells, including Tregs and TAMs, at right. Highly glycolytic tumor cells import glucose via GLUT1 and ferment it to lactate, which is coexported with protons into the TME via MCT1/MCT4. Glucose deprivation impairs the glycolytic capacity of Teff cells, which is key for their proliferation and translation of IFN-γ. Lactic acid impairs Teff cell proliferation by altering the NAD(H) redox balance. Utilization of lactic acid via MCT1 supports Treg proliferation and suppressive function. Lactic acid contributes to histone lactylation, which supports the expression of M2-like genes such as *Arg1* in macrophages. Tryptophan is depleted via IDO expressed by MDSCs, TAMs, and tumor cells. Tryptophan depletion triggers the stress response kinase GCN2 and suppresses the mTOR pathway, reducing proliferation, altering memory differentiation, reducing activation of Teff cells, and inducing a regulatory phenotype in naive T cells. Kynurenine, imported via SLC7A5/8, engages with the AhR to increase PD-1 and Foxp3 expression. Kynurenine-induced ROS inhibit IL-2 signaling critical for T cell survival. Depletion of oxygen in the TME inhibits oxidative metabolism and decreases the mitochondrial mass of CD8^+^ T cells. ROS both intra- and extracellularly drive partnerless nuclear factor of activated T cells (NFAT) signaling and expression of PD-1 and Tim-3 in CD8^+^ T cells while promoting NFAT/Foxp3 signaling in Tregs. Oxygen depletion promotes extracellular accumulation of ATP, which is broken down to adenosine by the cell-surface ectonucleotidases CD39 and CD73. Adenosine acts through A2AR to impair IL-2 and IFN-γ production and increase PD-1 expression in Teff cells, while activating Foxp3 and CTLA4 expression to promote the development of Tregs.

Tumor cells both deplete vital nutrients and produce “toxic” catabolites. From glucose to lactic acid, tryptophan to kynurenine, molecular oxygen to ROS, the tumor produces metabolites that shape immune cell function (Figure 1), and this may be therapeutically targeted. Here we review the metabolic landscape of the tumor from the perspective of metabolite abundance rather than scarcity, discussing how lactic acid, kynurenine, ROS, and adenosine shape immune cell function and how these are being targeted to improve immunotherapy.

## Lactic acid

A study analyzing metabolomics data from over 900 samples spanning seven different cancer types identified lactic acid as a consistently upregulated metabolite (7). Within the TME, lactic acid is derived from fermentation, by highly glycolytic tumor cells, of glucose to pyruvate and then, via lactate dehydrogenase (LDH), to lactate. Lactate and protons are then co-exported, producing extracellular lactic acid. In normal serum, lactate concentrations range from 1.5 to 3 mM (8), while tumor concentrations can range from 10 to 30 mM, reaching extremely high levels (50 mM) within necrotic tumor cores (9). Indeed, elevated levels of lactic acid indicate poor prognosis in several cancer types, including cervical (8), breast (10), head and neck (9, 11), high-grade gliomas (12), and non–small cell lung cancer (13). Therefore, it comes as no surprise that lactic acid, especially at tumor-equivalent concentrations, has profound effects on infiltrating immune cells (Figure 1). Emerging is lactate’s role as a major carbon source for many cell types in a homeostatic state (14). This is a prime example of how a toxic metabolite within the tumor is encountered in non-tumor contexts. Using infusions of ^13^C-labeled glucose and lactate into live mice, Hui et al. demonstrated that lactate, more than glucose, extensively contributes to the TCA cycle in all tissues examined (14). Indeed, even T cells, both effectors and Tregs, can utilize lactate to feed the TCA cycle (15, 16). By studying effector T cell metabolism through in vitro culture in glucose-rich media, the field has likely overestimated the importance of glycolysis for effector functions. In vivo carbon tracing has revealed that physiologically activated CD8^+^ T cells display an increased oxidative metabolism, in addition to a high glycolytic rate, compared with their in vitro–activated counterparts, opening the possibility that smaller carbon substrates like lactate play a larger role in effector CD8^+^ T cell metabolism than previously appreciated (17). These studies highlight the importance of lactate in shaping the metabolism of immune cells far before they enter the TME. Further detail regarding lactate–immune cell interaction in non-tumor tissues can be found in refs. 18–20. Understanding how immune cells metabolize in vivo under physiologic conditions will be key to developing successful metabolism-targeted therapies for the improvement of immunotherapy.

In general, lactate/lactic acid acts as an immunosuppressive metabolite. Indeed, activating effector CD8^+^ and CD4^+^ T cells in vitro in tumor-equivalent concentrations of lactic acid reduces their proliferation and capacity to produce cytokines (15, 16, 21). The presence of tumor-derived lactic acid acts as a double-edged sword, for where there is lactic acid there is glucose deprivation, both of which independently impact effector T cell function. As mentioned above, effector CD8^+^ and CD4^+^ T cells rely on glycolysis for translation of IFN-γ and proliferation (17, 22–25). While restricting glucose uptake helps promote CD8^+^ T cell memory formation, glucose is still critical for short-lived effector CD8^+^ T cells (26). However, tumor-infiltrating T cells experience glucose deprivation and lactic acid simultaneously. Several studies demonstrate that both lactate and lactic acid impair CD8^+^ and CD4^+^ effector T cell proliferation and cytokine production even in the presence of sufficient glucose (15, 16). Deeper mechanistic studies revealed that lactate works to limit T cell proliferation via the NAD(H) redox state, reducing NAD^+^ to NADH in lactate-rich conditions, resulting in altered NAD^+^-dependent enzymatic reactions and thus decreased glycolytic intermediates needed for proliferation (27). These data reveal a balance, as T cells are in constant contact with serum lactate and thus have a threshold for withstanding some lactate impairment. This threshold is likely determined at the level of lactate transport via solute carriers, like monocarboxylate transporter 1 (MCT1; encoded by *Slc16a1*). MCT1 is a bidirectional proton-assisted transporter with highest affinity for lactate (28). Directionality of transport is determined by both substrate and H^+^ ion concentration; thus T cells entering the TME likely experience an influx of lactic acid followed by changes in redox balance and impairment in proliferation and effector function, reducing tumor control (29). Notably, succinate, another MCT1 substrate and short-chain fatty acid (SCFA) (30, 31), can also be found at high levels within tumors and can mediate tumor-associated macrophage (TAM) polarization, metastasis, and angiogenesis (32, 33). Lactic acid’s and succinate’s comparable roles suggest that SCFAs act similarly within the TME.

Recent findings suggest that glucose may not be as limited in the TME as previously thought, meaning lactic acid could be contributing more to T cell dysfunction than lack of glucose (34). Pulsing mice bearing MC38 tumors (an immunogenic colon tumor model) with ^18^F-fluorodeoxyglucose (FDG), then measuring uptake directly ex vivo, revealed that tumor-infiltrating CD3^+^ T cells had FDG avidity similar to that of the MC38 cancer cells. While CD3^+^ T cells were not the highest FDG consumers, these data suggest that T cells have adequate access to and can compete for glucose within the TME. In addition, glycolysis was still occurring within the tumor and myeloid cell compartment, suggesting that T cells were exposed to lactic acid.

Not all immune cells respond negatively to tumor-derived lactic acid. The TME actively recruits and promotes the differentiation of Tregs, a subset of CD4^+^ T cells that express the transcription factor Foxp3 (35, 36). Tregs are potent suppressors of the immune system, tasked with maintaining immune homeostasis and preventing autoimmunity. Unlike effector cells, Tregs do not rely on glycolysis to meet their metabolic demands (15, 16, 37–39), but rely more heavily on oxidative metabolism, including lipid synthesis and signaling (40, 41). Their diminished glucose metabolism and reliance on alternative metabolites prime Tregs to thrive in the glucose-depleted TME and exert their immunosuppressive function. In contrast to effector T cells, lactic acid was shown to be critical for tumor-infiltrating Treg proliferation and function (16). Carbon tracing experiments revealed that lactic acid–supported proliferation was dependent on the generation of phosphoenolpyruvate, the starting intermediate for gluconeogenesis (16). Tumor-infiltrating Treg proliferation also likely relies on NAD(H) redox state, as Angelin et al. observed an increased NAD/NADH ratio in the presence of lactate in Foxp3^+^ induced Tregs compared with Foxp3^–^ conventional T cells (15). Additionally, lactate-influenced NAD/NADH ratio may play a role in the suppressive function of Tregs, as genetic impairment of complex I of the electron transport chain can lower NAD/NADH ratios and reduce Treg suppressive function (15). Consistent with these data is the observation that Treg-specific loss of the lactate transporter MCT1 reduced Treg-suppressive capacity and proliferation within the TME (16). Interestingly, Treg suppressive function positively correlated with the glycolytic activity of the tumors from which they were isolated, suggesting that lactic acid can enhance Treg suppressive capacity. Further research is needed to identify how NAD(H) redox balance in Tregs influences suppression and proliferation and whether this is the main mechanism by which lactic acid bolsters Tregs within the tumor.

Lactic acid also impacts innate immune cells. Lactic acid was found to polarize macrophages toward an M2-like/TAM-like state, including increased arginase 1 (Arg1) expression (42). Not only was lactic acid polarizing, but TAMs were found to have increased utilization of lactic acid. M2-like macrophages are known to be immunosuppressive (43), supporting the idea that cells that share a metabolism share a function. A potential mechanism for lactic acid’s influence on macrophage and Treg function may come through its contribution to histone lactylation and thus altered epigenetics (44). Zhang et al. identified that histones can undergo modification by lactylation, a histone mark with distinct dynamics compared with acetylation (44). Increasing histone lactylation late in M1 macrophage polarization resulted in increased Arg1 and other wound healing–associated gene expression, suggesting a shift to the immunosuppressive M2 macrophage phenotype. Lactylation may play a similar role in Tregs to induce or fortify the expression of immunosuppressive genes. Further research into lactate utilization by immune cells and its ability to drive a suppressive phenotype is warranted.

## Kynurenine

Another metabolite consistently upregulated across multiple tumor types is kynurenine (7). Like lactic acid, kynurenine is an immunosuppressive by-product derived from the depletion of a critical metabolite, in this case tryptophan. Tryptophan is one of nine essential amino acids required by humans and plays roles in protein synthesis, serotonin production, and immune cell regulation (45, 46). Depletion of tryptophan and production of kynurenine are driven by three rate-limiting enzymes, indoleamine 2,3-dioxygenase 1 (IDO1), IDO2, and tryptophan 2,3-dioxygenase (TDO). Many studies have focused on IDO1 as the main contributor to tryptophan depletion because TDO tissue expression is relatively restricted (47–49), and while IDO2 is widely expressed, it has a reduced capacity for breaking down tryptophan and its role in inflammation is debated (50–53). IDO1 is expressed by many cell types, including immune cells, epithelial cells, cancer cells, and fibroblasts. IDO1 expression is greatly enhanced by IFN-γ generated during tissue inflammation and acts as a negative-feedback loop to curb excessive inflammation (54–56). This explains the paradoxical expression of IDO1 on some proinflammatory cells such as M1 macrophages (57).

Beyond the tumor, kynurenine plays a role in shaping the immune cell function. Kynurenine was found to be key in regulating maternal-fetal tolerance, as pharmacologic inhibition of IDO in mice resulted in maternal T cell–mediated rejection of allogeneic fetuses (58). In addition, kynurenine plays a role in the maintenance of immune-privileged sites, such as the eyes and the brain (reviewed in ref. 45). While the TME takes advantage of the immunosuppressive nature of IDO1 and kynurenine, tumors did not “patent” this mechanism, but rather utilized what evolved to maintain immune privilege. This perspective is critical as new in vivo techniques shift our understanding of the TME metabolic landscape and we identify new or underappreciated metabolites with a large physiologic role in shaping immune function (17, 34).

Like glucose and lactic acid, tryptophan depletion and kynurenine production have independent immunosuppressive effects (Figure 1). Depletion of tryptophan triggers the stress response kinase general control nondepressible 2 (GCN2) and suppresses the mTOR pathway, resulting in reduced proliferation and activation of effector T cells as well as inducing a regulatory phenotype in naive T cells (59, 60). Independently, kynurenine can suppress through several mechanisms, including (a) promotion of tolerogenic antigen-presenting cell differentiation (61, 62), (b) promotion of Treg differentiation via the aryl hydrocarbon receptor (AhR) (63, 64), and (c) inhibition of IL-2 signaling (65). It is well established that many tumor types express IDO1, with high expression associated with poor prognosis and increased presence of tumor-infiltrating Tregs (66–70). Kynurenine can also have a direct impact on effector T cells, as T cell receptor (TCR) stimulation can increase kynurenine uptake via *Slc7a5*/*Slc7a8*, leading to increased programmed cell death 1 (PD-1) expression induced by AhR ligation (71, 72). Notably, many in vitro experiments use much higher concentrations of kynurenine to impair T cells than what is found in vivo (73, 74), putting into question its clinical significance. However, the kynurenine pathway produces several different metabolites, with kynurenine itself able to be further metabolized, making it difficult to understand kynurenine’s full impact in vivo. Treating mice with a specific kynurenine-depleting enzyme improved tumor growth and enhanced immunotherapy, indicating that in vivo concentrations of kynurenine may still be clinically relevant (74). Ultimately, lack of tryptophan and production of kynurenine impair activation of tumor-infiltrating effector T cells that are critical for clearing tumors and promote the presence of immunosuppressive Tregs. Tryptophan depletion and kynurenine production are employed to create an immunosuppressive environment, which at steady state maintains immune tolerance but in tumors is exploited to evade immune destruction.

## ROS and adenosine

Many tumors, if not all, experience some level of oxygen depletion (hypoxia) (75). Oxygen depletion occurs when the poor vascularization and high metabolic demand of the tumor outpace the available oxygen supply. As with glucose and tryptophan depletion, oxygen depletion is accompanied by the production of toxic by-products such as ROS and adenosine, which have been a heavy focus in cancer research (76, 77). ROS are produced as a normal part of oxidative metabolism and are important for normal cell survival, signaling, and homeostasis (78). However, cancer takes advantage of ROS, using their overproduction, among other things, to drive mitogenic signaling pathways, metastasis, and survival (79, 80). In addition to ROS, tumor hypoxia drives the accumulation of extracellular ATP, which is broken down to the immunosuppressive metabolite adenosine (81, 82). Primarily, ATP release and adenosine generation act on purinergic receptors to impair immune cell infiltration and activation, thus decreasing antitumor immunity (83, 84). While tumor-derived ROS and adenosine have been extensively investigated, there remains a deep interest in understanding and manipulating their impact on immune cells to improve cancer therapies.

As with lactic acid and kynurenine, ROS play an important role in shaping immune cell function in non-tumor contexts. For example, upon encounter with microbial invaders, innate immune cells utilize NADPH oxidase–derived (NOX-derived) superoxide to disrupt iron-sulfur centers and kill microorganisms (85). ROS can also act in chemotaxis, signaling neutrophils and other immune cells to sites of injury or infection, and even activate and mature dendritic cells (86–89). In fact, even T cell activation requires some level of mitochondrial ROS (mROS) production (90). In contrast, ROS can also play an antiinflammatory role, as ROS produced by antigen-presenting cells are critical for suppressing autoreactive T cells in a model of arthritis (91, 92). In humans, mutations in NOX2 lead to chronic granulomatous disease, in which recurrent and severe infections are associated with an increased and prolonged inflammatory gene profile in neutrophils compared with healthy controls (93). These findings highlight that innate and adaptive immune systems are tuned by ROS long before they enter the TME. What make the TME distinct are the continuous and high levels of ROS in comparison with normal tissue, pushing infiltrating immune cells to respond at the extreme end of their previous attunement.

Like other toxic by-products discussed here, high levels of ROS impair effector T cells within the TME (Figure 1). The metabolic fitness and antitumor efficacy of CD8^+^ T cells rely heavily on oxidative metabolism. CD8^+^ T cells that infiltrate the TME undergo a loss of mitochondrial mass and decrease of cytokine production, which can be rescued by overexpression of the regulator of mitochondrial biogenesis PPARγ coactivator 1α (PGC1α) (94). Further, more oxidative (thus more hypoxic) TMEs are associated with increased CD8^+^ T cell exhaustion and decreased response to anti–PD-1 immunotherapy (95, 96). Recent mechanistic insight revealed that continuous TCR stimulation and hypoxia drive increased mROS production by CD8^+^ T cells, which is sufficient to induce an exhaustion-like phenotype (97). Overexpression of Gpx1, a glutathione peroxidase capable of acting on a variety of ROS, reduced ROS accumulation and increased IFN-γ production by tumor-infiltrating CD8^+^ T cells. These data are consistent with T cell response to macrophage-derived ROS, as this also impairs IFN-γ production (91). In addition, myeloid-derived suppressor cells (MDSCs), an abundant suppressive cell population within tumors, can impair antigen recognition by CD8^+^ T cells in a ROS-dependent manner (98).

In addition to impairing effector cells, high levels of ROS can support regulatory populations (Figure 1). Some evidence suggests that macrophage-derived ROS may induce the formation of peripheral Tregs (99). Consistent with this is the finding that ROS, including tumor-derived ROS, can trigger the accumulation of SUMO-specific protease 3 (SENP3), a protein crucial for deSUMOylation of BACH2, in Tregs, which results in the repression of genes associated with effector CD4^+^ T cells (100). In addition to Tregs, MDSCs represent a major immunosuppressive population within the TME. Like Treg formation, MDSC formation appears to be supported by oxidative stress–prone tissues (101). MDSCs display a high level of oxidative metabolism, which is key to their suppressive capacity (102). Nuclear factor (erythroid-derived 2)–like 2 (Nrf2), a key antioxidant protein, was identified as a key player in promoting and maintaining MDSC identity by balancing redox levels within the cell (102). MDSCs also utilize ROS to suppress effector T cell responses. In particular, expression of NOX2, a generator of extracellular ROS, was found to be required on MDSCs for the suppression of T cells and maintenance of MDSC identity (103). Taken together, these findings suggest that ROS are vital for normal immune functioning at low levels but, at high levels in the TME, promote the dysfunction of effector cells and the presence of regulatory populations.

Adenosine is a potent immunosuppressive metabolite that both impairs effector cells and supports regulatory cells (Figure 1). Within the TME, adenosine is derived via the cell surface ectonucleotidases CD39 and CD73 expressed by both tumor cells and infiltrating immune cells. Hypoxia drives HIF1A activity, which in turn upregulates CD39, CD73, and the adenosine receptor A2BR (104–106). Extracellular ATP is converted to ADP and/or AMP by CD39, while AMP is converted to adenosine by CD73 (77). Adenosine then binds to one of four receptors, A1R, A2AR, A2BR, or A3R, to exert its regulatory functions. While A1R, A2AR, and A3R have high affinity for adenosine, A2AR and A2BR signal through cyclic AMP (cAMP), which is associated with profound immunosuppression (107–109). Signaling through A2AR and A2BR can decrease IFN-γ and IL-2 production and increase the inhibitory molecule PD-1 in effector cells, and can activate Foxp3, CTLA4, and Lag3, promoting the development of Tregs (77).

It is important to note that coexpression of CD39 and CD73 on the same cell is not required to produce adenosine. While cells such as Tregs, regulatory dendritic cells, and tumor cells can certainly express both CD39 and CD73, it may be that one cell expresses CD39 while a neighboring cell expresses CD73, inducing local adenosine production (77). This may be the case with exhausted CD8^+^ T cells, which express high amounts of CD39 (110). High expression of CD39 may confer a suppressive capacity to exhausted T cells by creating a pool of AMP that can be converted to adenosine by the T cells themselves or by neighboring cells expressing CD73. Indeed, production of adenosine is a known suppressive tactic of Tregs (111); therefore, research into the immunosuppressive capacity of exhausted T cells is warranted.

Notably, all the toxic by-products discussed here tend to negatively impact effector cells while supporting regulatory immune cells. This suggests that regulatory immune cell populations have evolved to be metabolically “out of synch” with their effector cell counterparts, utilizing the metabolites effector cells and inflamed tissues produce to reign in immune responses and prevent tissue damage. Selective pressures on the TME have pushed tumors to utilize these toxic by-products to maintain immune tolerance. While each toxic by-product is not unique to the TME, the combination and high production of these by-products make it distinct.

## Implications for immunotherapy

Recognition of the abundance and immunomodulatory impacts of lactic acid, kynurenine, ROS, and adenosine has led to the development of therapies targeting their production in the hope of improving cancer immunotherapies. The benefit of targeting these toxic metabolites is their abundance across multiple tumor types, offering a broad-range therapy. However, it will be important to understand how inhibition of lactic acid, kynurenine, or ROS will interact with a range of immunotherapies. Further understanding of how these toxic metabolites can be limited to alter immune cell function will help the field utilize the proper immunotherapy to achieve maximal efficacy. Here we approach metabolic therapies from two perspectives: altering the metabolic landscape of the tumor versus altering the metabolism of infiltrating immune cells to overcome the TME.

### Altering the metabolic landscape of the tumor.

**Figure 2:**
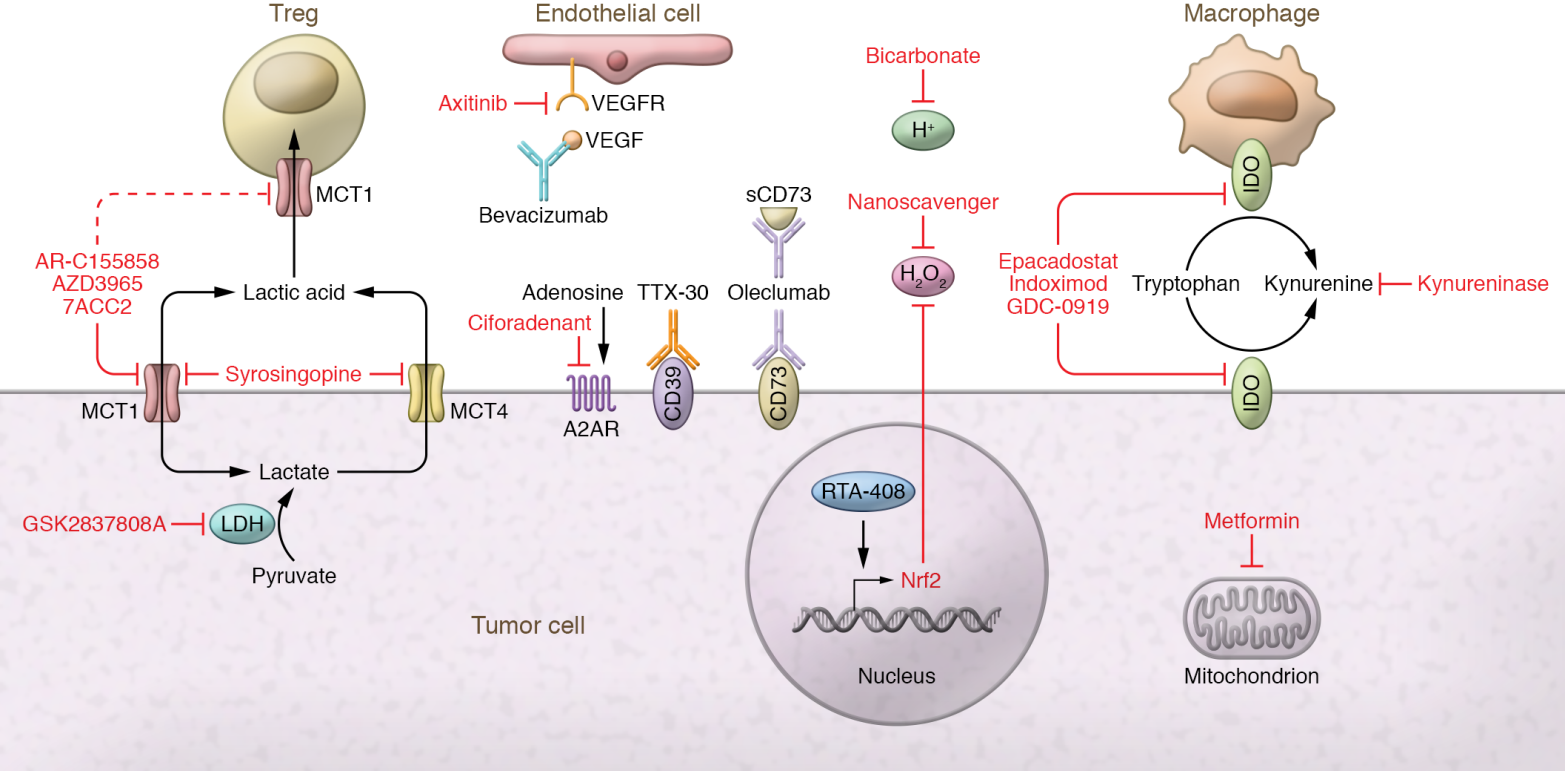
Metabolic alteration of the TME to improve cancer immunotherapy. Alteration of the metabolic landscape of the tumor can be accomplished in many ways. Lactic acid production by tumor cells can be targeted using an inhibitor of LDH (GSK2837808A). Alternatively, lactic acid export by tumor cells could be targeted using MCT1 inhibitors (AZD3965 in clinical trials, 7ACC2 and AR-C155858 in preclinical work) or MCT4/MCT1 dual inhibitors (syrosingopine). MCT1 inhibitors may also block Treg import and usage of lactic acid, leading to diminished suppressive function and proliferation. Lactic acid lowers the pH of the TME, which can be counteracted through bicarbonate treatment. Tryptophan depletion and kynurenine production can be targeted by inhibition of IDO found on tumor cells and TAMs (epacadostat, indoximod, GDC-0919). Alternatively, kynurenine alone can be depleted using an enzyme engineered for its degradation (PEGylated kynureninase). Oxygen depletion can be targeted using VEGF inhibitors (bevacizumab) or VEGFR inhibitors (axitinib) to normalize tumor vasculature and improve tumor oxygenation. Metformin, a common diabetes drug, can be used to decrease tumor hypoxia, potentially through its action as a mitochondrial complex I inhibitor. ROS can be targeted through drugs promoting endogenous ROS scavengers (RTA-408 promoting Nrf2) or by addition of exogenous engineered ROS nanoscavengers. The production of adenosine can be targeted using monoclonal antibodies against CD39 (TTX-30) and CD73 (both membrane bound and soluble [sCD73]; oleclumab) or by small-molecule inhibition of the A2AR (ciforadenant).

Many strategies exist for altering the metabolic landscape of the tumor (Figure 2). For example, lactic acid production can be targeted by inhibiting lactate dehydrogenase (LDH), the enzyme responsible for converting pyruvate to lactate, or inhibiting glycolysis at an earlier point in the pathway. LDH levels in the blood and TME are associated with poor outcomes for cancer patients and are used in determining tumor staging in melanoma (112). For melanoma patients, high LDH levels are predictive of poor response to anti–PD-1 immunotherapy (113, 114). To date, glycolytic inhibitors are still in the preclinical phase, so their impact on human patients is unknown. Despite this, preclinical models provide compelling evidence that blocking lactic acid production enhances immunotherapy. One study demonstrated that knocking down LDHA in 4T1 and B16 tumors increased response to anti-CTLA4 treatment, in part by shifting Tregs away from lactate metabolism and increasing Treg glucose uptake (16, 115). Using a pharmaceutical approach, another study showed that inhibition of patient-derived and B16 melanoma LDHA with the inhibitor GSK2837808A enhanced T cell killing both in vitro and in vivo and enhanced adoptive cell therapy (116). While specific LDH and glycolytic inhibitors have not fully made it into the clinic, we could potentially repurpose old drugs that have glycolysis-inhibiting effects. For example, diclofenac, a common NSAID, has been shown to modulate glycolysis independent of COX inhibition and could be used to improve anti–PD-1 immunotherapy (117, 118). While we have focused our discussion on immune cells, we appreciate that inhibiting tumor cell glycolysis directly impacts the fitness of tumor cells. Therapeutically, this is ideal, as inhibiting tumor cell glycolysis can both kill tumors and promote immune cells. A more detailed discussion on how inhibiting glycolysis impacts the fitness of tumor cells can be found in ref. 119.

Lactic acid can also be decreased within the TME by targeting of its export. Lactic acid is transported via MCTs (28). MCT1, almost ubiquitously expressed, has the highest affinity for lactate and can both import and export lactate based on the concentration gradient of substrate and protons. MCT4 is more heavily expressed by highly glycolytic tissues, including tumor cells, and while it is also a bidirectional transporter, it mainly participates in the export of lactate (28). While many small-molecule inhibitors of MCT1 and MCT4 have been developed for preclinical use, only AstraZeneca’s AZD3965 compound is currently being tested for use in humans (ClinicalTrials.gov NCT01791595). Preclinical work has shown that AZD3965 can lower lactic acid secretion into the TME and increase tumor immune cell infiltration (120). However, these findings should be taken cautiously, as data were generated from Raji xenograft–bearing SCID mice and pertained only to NK and myeloid cells. In fact, use of immunodeficient models is a limitation of many preclinical studies using MCT inhibitors, and further work on how MCT inhibitors impact adaptive immune cells, especially within the TME, is warranted (121–123). In light of this, deletion of MCT1 specifically on Tregs was shown to slow tumor growth and synergize with anti–PD-1 therapy, suggesting that pharmacologic inhibition of MCT1 may play a dual role, both to inhibit lactate secretion by tumor cells and to impair immunosuppressive Tregs (16).

Lactic acid creates a low-pH environment, which can be targeted to improve cancer therapy. To counteract acidity, bicarbonate has been used, administered by a variety of methods (reviewed in ref. 124). Bicarbonate administration has been shown to control Yumm1.1 melanoma growth and increase CD8^+^ T cell infiltration and NK and B cell activation, as well as improve anti-CTLA4 and anti–PD-1 therapy and adoptive cell therapy, in B16 melanoma–bearing mice (125, 126). In addition, bicarbonate can act to alter cancer cell mTORC signaling, which may help to limit lactic acid production (127).

Kynurenine metabolism can also be targeted at several points (Figure 2). Much of the work targeting kynurenine has been through the inhibition of IDO1 (128). Theoretically, in the context of the TME, inhibition of IDO1 is the best of both worlds, stopping tryptophan depletion and kynurenine production. Indeed, preclinical models targeting IDO1 strongly enhanced B16 and 4T1 tumor response to both anti-CTLA4 and anti–PD-1 therapy, and demonstrated efficacy regardless of tumor IDO1 expression (129, 130). As a result, several IDO inhibitors are in clinical trials (NCT04049669, NCT03432676, NCT02471846). Unfortunately, Incyte’s trial of epacadostat in combination with pembrolizumab (anti–PD-1) was stopped after intermediary analysis revealed no added benefit of IDO1 inhibition (NCT03432676). While this has certainly dampened enthusiasm for targeting IDO1, it highlights the complexity of targeting the IDO pathway. It may be that some level of tryptophan catabolism is required to create an optimally tuned antitumor immune response. Lack of tryptophan catabolism could lead to a buildup of serotonin, which has been shown to have protumor effects and modulate immune cell function and mitochondrial metabolism (131, 132). While preclinical models suggested synergy between IDO inhibition and checkpoint blockade, in humans a better synergy may be found among chimeric antigen receptor (CAR) T cell therapy, oncolytic viruses, and/or cytokine treatment. More work is needed to identify whether IDO1 inhibitors will truly boost immunotherapies.

Kynurenine can also be targeted directly, leaving IDO and tryptophan catabolism intact. Using a pharmacologically optimized enzyme, PEGylated kynureninase, one study demonstrated that peritumoral injection slowed B16 and CT26 tumor growth in a CD8^+^ T cell–dependent manner (74). Administration of PEGylated kynureninase improved the efficacy of anti–PD-1 therapy in B16 tumors, anti-CTLA4 in 4T1 tumors, and a cancer vaccine in CT26 tumors. These findings suggest that kynurenine plays a larger role than tryptophan depletion in inhibiting antitumor immune response, supporting the idea that the toxic by-products, rather than the depletion of key nutrients, drive metabolic immunosuppression in the TME.

Owing to the variety of ROS-generating mechanisms, ROS production can be targeted in many ways (Figure 2). One promising method is through the reduction of tumor hypoxia. In preclinical models, metformin, a common type 2 diabetes drug that can act as a weak mitochondrial complex I inhibitor, reduced tumor hypoxia and promoted B16 tumor clearance when combined with anti–PD-1 (96). While not statistically significant, a retrospective cohort study revealed a trend toward improved outcomes of melanoma patients on metformin being treated with checkpoint blockade (133). Tumor hypoxia can also be targeted through the inhibition of VEGF. In an attempt to oxygenate the TME, tumors secrete VEGF and promote angiogenesis, but the blood vessels formed are highly disorganized and leaky, providing inadequate oxygen (1, 134, 135). Low doses of antiangiogenic antibodies (anti-VEGFR2) have been shown to normalize the tumor vasculature, which improves M1-like macrophage polarization, T cell tumor infiltration, and whole-cancer-cell vaccine therapy in murine breast cancer models (136). In addition, many others (reviewed in ref. 135) have shown in preclinical models that antiangiogenic treatments can improve anti–PD-L1 therapy. Clinically, antiangiogenic and immunotherapy combinations have shown the best efficacy in renal cell carcinoma (RCC) and hepatocellular carcinoma (HCC). Patients with previously untreated metastatic RCC were given pembrolizumab and axitinib (VEGFR1, VEGFR2, and VEGFR3 inhibitor) combination therapy, which statistically improved progression-free survival over sunitinib monotherapy, leading to FDA approval for treatment-naive RCC patients (137). Likewise, combination of atezolizumab (anti–PD-L1) and bevacizumab (anti–VEGF-A) led to improvements in progression-free and overall survival compared with standard of care in unresectable HCC patients (138).

Another way tumor-derived ROS can be targeted is with the use of scavengers. ROS production in the TME is finely tuned by ROS generators and ROS scavengers to maintain optimal levels for signaling, growth, and survival (80). However, this finely tuned balance can be disrupted by addition of exogenous ROS scavengers or induced expression of endogenous ones. One group developed an extracellular matrix–targeting, pH-sensitive ROS nanoscavenger that can target to the TME, decrease ROS, and improve antitumor immunity in several tumor models (139). This study provides proof of principle for use of a manufactured ROS scavenger in the improvement of immunotherapy. Alternatively, endogenous ROS scavengers could be induced, for example using the drug RTA-408, also known as omaveloxolone. RTA-408 was shown to induce Nrf2, a major protein involved in oxidative stress protection, and resulted in suppression of ROS in tumor xenograft models (140–142). In 2019, a phase Ib/II clinical trial was completed combining anti-CTLA4 and anti–PD-1 with RTA-408 in melanoma patients, but results have not been formally posted (NCT02259231).

In addition to the hypoxia-reducing methods above, adenosine production can be targeted directly. These drugs take the form of small-molecule inhibitors or blocking antibodies mainly targeting CD73, CD39, and A2AR (reviewed in ref. 143). While these drugs have shown preclinical efficacy in reducing adenosine production and even preventing the ectonucleotidase of soluble forms of CD73 (MEDI9447, also known as oleclumab; ref. 144), most are awaiting results from phase I/II clinical trials (143). Interestingly, intraperitoneal treatment of tumor-bearing mice with antisense oligonucleotides targeting CD39 augmented CD8^+^ T cell proliferation, reduced CD39 expression by tumor and Tregs, and enhanced anti–PD-1 treatment (145). These results are encouraging, and hopefully a similar efficacy will be achieved in human trials.

While we have discussed targeting of a single toxic metabolite in combination with immunotherapy, we appreciate that many tumors undergo multiple metabolic changes and thus produce a collection of toxic metabolites. Thus, it may be necessary to target several sources of toxic metabolites in combination with immunotherapy to yield the best therapeutic efficacy.

### Altering the metabolism of infiltrating immune cells.

**Figure 3:**
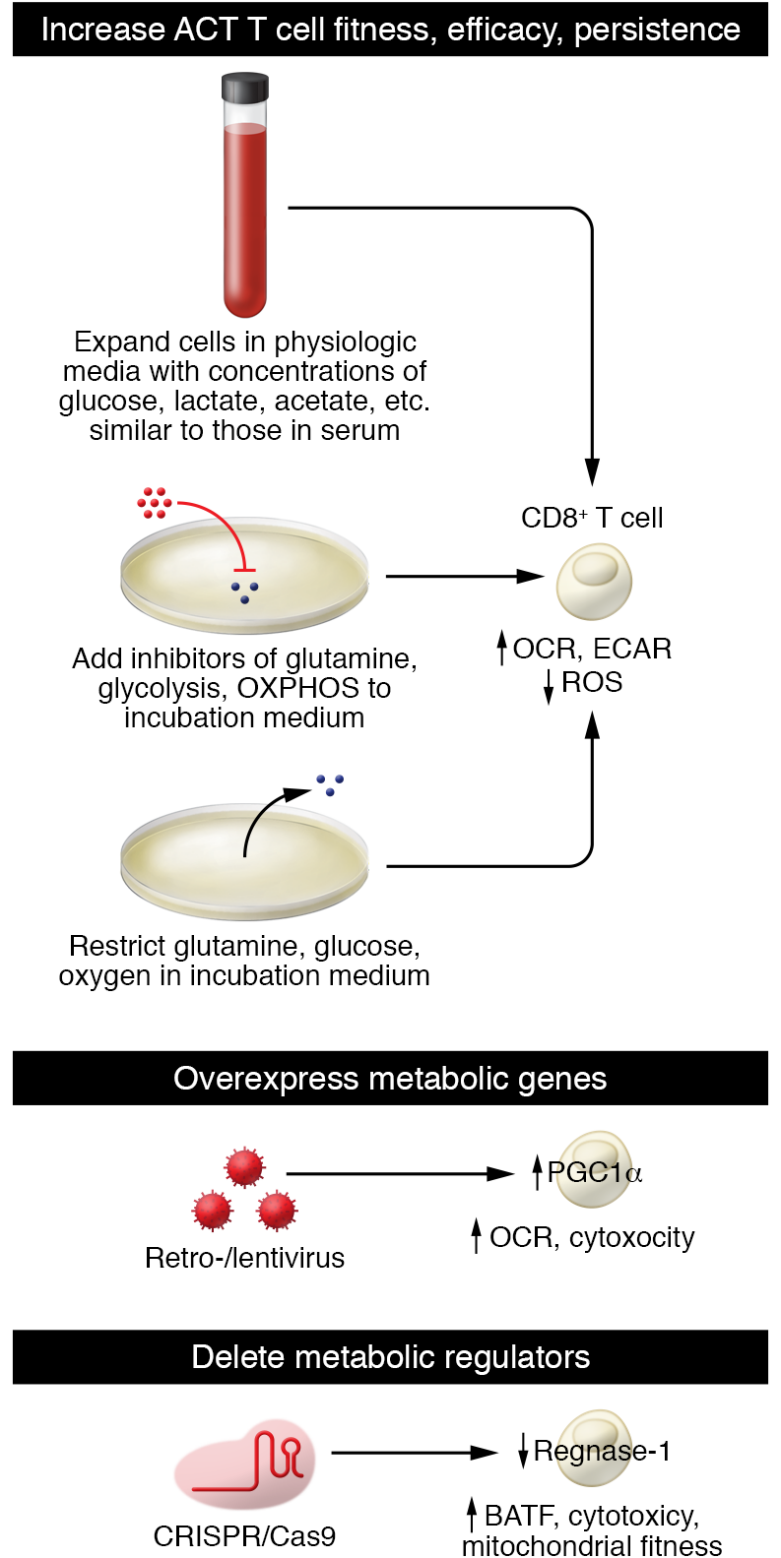
Metabolic bolstering of T cells to better withstand the TME. Instead of altering the TME, T cells used for cellular therapies (CAR T cells or adoptive cell therapy) can be metabolically bolstered before patient reinfusion. During the in vitro expansion phase of cellular therapies, limiting metabolites such as glucose, glutamine, or oxygen in the media or using a medium with physiologic metabolite concentrations may better prepare T cells for survival and efficacy in the metabolically harsh TME. Alternatively, T cells can be engineered to either overexpress key metabolic genes, such as *PGC1a*, to improve mitochondrial fitness, or delete metabolic regulators such as regnase-1, which negatively regulates mitochondrial fitness, to give T cells a metabolic edge within the TME.

CAR T and adoptive T cell therapies provide a way to metabolically bolster T cells to function in the harsh TME (Figure 3). These therapies require taking a patient’s blood, enriching it for T cells, then activating, expanding, and (in some cases) genetically altering them via viral or nonviral methods. In vitro expansion and transduction provide a window in which T cell functioning can be enhanced to better compete in the TME. One method of bolstering CAR T cells is to overexpress or delete genes that regulate metabolism. For example, overexpression of PGC1α, a transcriptional coactivator key for mitochondrial biogenesis, prevented the loss of mitochondrial mass and improved antitumor efficacy in an adoptive cell therapy model (94). Conversely, deletion of *Regnase-1*, a gene identified as negatively regulating the transcription factor BATF and mitochondrial metabolism in CD8^+^ T cells, improved the efficacy of adoptive cell transfer (146). While these are two targets of many, they represent a means of genetically altering T cells to improve efficacy against solid tumors.

Adoptive T cell therapies can also be metabolically bolstered through their expansion media. Commonly used media such as RPMI, DMEM, and AIM V contain high amounts of glucose and reduced metabolite levels compared with serum, poorly preparing them for the harsh metabolic landscape of the tumor (147). As T cells are highly sensitive to their metabolic environment (17, 148), expansion of T cells in media containing or lacking certain metabolites may improve their persistence and efficacy in vivo. Consistent with this, glutamine restriction in vitro by either nutrient starvation or metabolic inhibitor enhanced the efficacy of adoptively transferred T cells in mice (149). Restricting metabolites or adding metabolic inhibitors to expansion media is an attractive method of improving adoptive cell therapies because of its relative simplicity, making it a hot area of investigation.

## Conclusions

Tumors not only consume essential metabolites but simultaneously produce toxic by-products, which persist in the TME because of its poor perfusion. Both the consumption of metabolites such as glucose, amino acids, and oxygen and the production of lactic acid, kynurenine, ROS, and adenosine negatively regulate effector immune cells and support regulatory immune populations. While the TME is not unique in its production of these toxic metabolites, it is distinct with its high persistent levels of them. As we further investigate immunometabolism in the TME and how best to modulate it to improve immunotherapy, it is critically important to remember that depletion and production of metabolites both have independent impacts on immune cell function. As in the failed IDO1 inhibitor trial, there may be a more finely tuned balance between depletion of essential metabolites and production of toxic ones than we appreciate. Ultimately, understanding the physiologic balance between essential metabolites and their toxic by-products and the subsequent impact on immune cells will be key to developing approaches to fuel curative immunotherapy for cancer.

## Version 1 01/18/2022

Electronic publication
